# Low annual temperature likely prevents the Holarctic amphipod *Gammarus lacustris* from invading Lake Baikal

**DOI:** 10.1038/s41598-021-89581-x

**Published:** 2021-05-18

**Authors:** Kseniya Vereshchagina, Elizaveta Kondrateva, Andrei Mutin, Lena Jakob, Daria Bedulina, Ekaterina Shchapova, Ekaterina Madyarova, Denis Axenov-Gribanov, Till Luckenbach, Hans-Otto Pörtner, Magnus Lucassen, Maxim Timofeyev

**Affiliations:** 1 Institute of Biology, Irkutsk State University, Karl Marx str.1, Irkutsk, 664003 Russia; 2 Department of Integrative Ecophysiology, Alfred Wegener Institute Helmholtz Centre for Polar and Marine Research, Am Handelshafen 12, 27570 Bremerhaven, Germany; 3 Irkutsk State University, Karl Marx str.1, Irkutsk, 664003 Russia; 4 Department of Bioanalytical Ecotoxicology, UFZ-Helmholtz Centre for Environmental Research, Permoserstr. 15, 04318 Leipzig, Germany

**Keywords:** Ecophysiology, Invasive species

## Abstract

Species with effective thermal adaptation mechanisms allowing them to thrive within a wide temperature range can benefit from climatic changes as they can displace highly specialized species. Here, we studied the adaptive capabilities of the Baikal endemic amphipods *Eulimnogammarus verrucosus* (Gerstfeld, 1858) and *Eulimnogammarus cyaneus* (Dybowsky, 1874) compared to the potential Holarctic Baikal invader *Gammarus lacustris* Sars, 1863 at the cellular level including the energy metabolism and the antioxidant system. All species were long-term exposed to a range of temperatures (1.5 °C to mimic winter conditions and the three species-specific preferred temperatures (i.e., 6 °C for *E. verrucosus*, 12 °C for *E. cyaneus* and 15 °C for *G. lacustris*). At 1.5 °C, we found species-specific metabolic alterations (i.e., significantly reduced ATP content and lactate dehydrogenase activity) indicating limitations on the activity level in the Holarctic *G. lacustris*. Although the two Baikal endemic amphipod species largely differ in thermal tolerance, no such limitations were found at 1.5 °C. However, the cold-stenothermal Baikal endemic *E. verrucosus* showed changes indicating a higher involvement of anaerobic metabolism at 12 °C and 15 °C, while the metabolic responses of the more eurythermal Baikal endemic *E. cyaneus* may support aerobic metabolism and an active lifestyle at all exposure temperatures. Rising temperatures in summer may provide a competitive advantage for *G. lacustris* compared to the Baikal species but the inactive lifestyle in the cold is likely preventing *G. lacustris* from establishing a stable population in Lake Baikal.

## Introduction

Temperature is one of the most pervasive abiotic factors directly affecting all levels of organismic organization of aquatic ectotherms. Species with high phenotypic plasticity that are equipped with mechanisms to thrive within a wide thermal window may benefit from environmental change as they may displace less adapted species^1^. For example, invasive species often characterized by high phenotypic plasticity can displace populations of native species that are closely adapted to the habitat-specific conditions. Especially in regions subjected to significant climate changes including warming and temperature extremes, this can lead to a shift in species composition with critical consequences for aquatic ecosystems^2–4^.

Ancient ecosystems like Lake Baikal are mostly inhabited by highly specialized and often endemic species^5^. Endemic species are often narrowly adapted to specific environmental conditions and particularly sensitive to the negative effects of climate change^6^. Unraveling the physiological mechanisms of thermal adaptations is key for understanding and predicting the impact of global climate change on ecosystems^7–9^. Lake Baikal is one of the most striking examples of ancient lakes with a diverse and at the same time potentially vulnerable flora and fauna. The lake is about 25–30 million years old^10^. It is characterized by high biodiversity (about 2595 species), a large proportion of endemic species (about 80%) and is also considered one of the most famous and important centers of endemic speciation among freshwater ecosystems^11^. Amphipods (Amphipoda: Crustacea) are one of the most important taxonomic groups of Lake Baikal’s benthic fauna; they dominate in terms of abundance, diversity (350 species and subspecies) and biomass^12^.

The Baikal ecosystem is characterized by the unique phenomenon called “the relative immiscibility” between Baikal and Palearctic faunas ^13^. This term describes the limited invasion of Holarctic and Palearctic fauna into Lake Baikal and, at the same time, limited distribution of Baikal species outside of Lake Baikal^14^. It is assumed that the long evolutionary history of the lake isolated from other water reservoirs caused the formation of a highly specialized fauna exploiting all available resources in Lake Baikal and successfully outcompeting invaders^13^. One important adaptation is the ability of Baikal littoral endemic amphipods to maintain high physiological activities down to 0 °C and—for some species—to reproduce under ice at these temperatures^15,16^. The vast majority of the Baikal fauna is cold stenothermal and does not tolerate rapid warming within the littoral zone in summer ^14,17^. Climate change alters the lake’s temperature regime, causing shorter winters^18^ and more frequent extreme heat events in summer^19,20^. This may cause a mismatch between highly adapted species and their respective niche, causing a disruption of the lake’s immiscibility barrier.

We previously found differences in thermal tolerance and thermal preferences between the winter-reproducing Baikal endemic littoral amphipod species *Eulimnogammarus verrucosus* (Gerstfeld, 1858), the summer-reproducing Baikal endemic species *E. cyaneus* (Dybowsky, 1874), and the Holarctic *Gammarus lacustris* Sars, 1863 inhabiting adjacent water bodies. *G. lacustris* is considered a potential Lake Baikal invader^6,21,22^. Different strategies of metabolic and behavioral adaptations of the Baikal endemic cold stenothermal *E. verrucosus*, the Baikal endemic thermotolerant *E. cyaneus* (Dybowsky, 1874), and the ubiquitous non-Baikal species *G. lacustris* species towards warming could be shown^21,23^. Thus, *E. verrucosus* shows a behavioral response by migrating to deeper water in the littoral zone when the temperature surpasses its rather low metabolically critical threshold in the summer^21^. *Eulimnogammarus cyaneus*, in contrast, tolerates increased temperatures in the summer by investing resources into the cellular chaperone system^24^. While the cold stenothermal amphipod fauna of Baikal may be stressed in summer by increased temperatures in the littoral, which could be disadvantageous, their ability to maintain a high metabolic activity in winter may outcompete Holarctic species like *G. lacustris*, which significantly reduces its metabolic activity during the winter^25^. The superiority of Baikal endemic amphipods at low temperature may thus prevent the non-Baikal species to colonize the lake.

We hypothesize that the Baikal amphipod species have metabolic adaptations of the energy metabolism to deal with cold temperatures (i.e., cold-compensated enzyme activities due to higher densities of mitochondria in cold stenotherms) to maintain the generation of energy equivalents at low temperatures. To test this hypothesis, we aimed at investigating metabolic activity and cellular energy state as well as the activity of the antioxidant system as a marker of cellular stress in the littoral benthic amphipods *E. verrucosus* and *E. cyaneus* from the Baikal littoral, and non-Baikal Holarctic *G. lacustris* after long-term cold exposure (1.5 °C) compared to a range of temperatures, including the respective species-specific preferred temperature.

**Figure 1:**
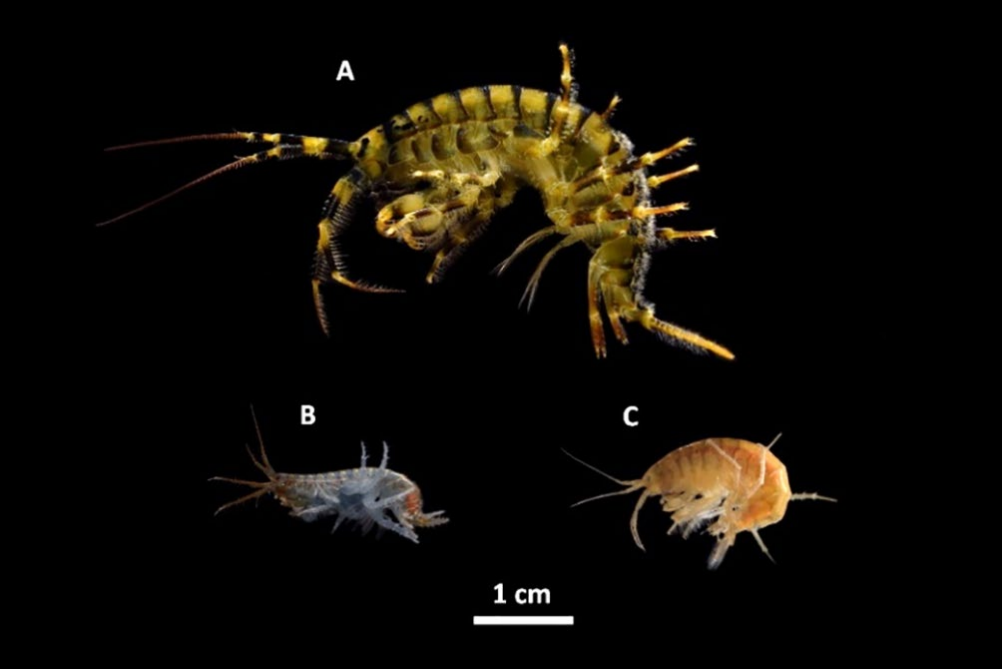
Photographs of adult individuals of the Baikal endemic amphipod species *Eulimnogammarus verrucosus* (Gerstfeldt, 1858) (**A**) and *Eulimnogammarus cyaneus* (Dybowski, 1874) (**B**) and *Gammarus lacustris* Sars,1863 (**C**), occurring across the Holarctic.

The here investigated Baikal species are the most dominant amphipods in the littoral zone of the lake. The amphipod *G. lacustris* is widely distributed across the Holarctic and occurs in waters connected to Lake Baikal, but not in the open Baikal (Fig. 1).

In this long-term cold acclimation pilot study, we focused on central aspects of the energy metabolism—the biochemical characteristics of aerobic and anaerobic energy generation and cellular energy storages. The cellular energy state was estimated by quantifying ATP, the main energy equivalent of a cell, glucose as a readily available metabolic fuel and glycogen—as the storage of glucose in cells. Additionally, activities of key enzymes of the aerobic metabolism—cytochrome c oxidase and citrate synthase were measured. Cytochrome c oxidase is the enzyme of the respiratory electron transport chain, which is responsible for aerobic ATP-generation. Citrate synthase catalyzes the first step in the citric acid cycle, which represents the central hub for the metabolism of carbohydrates, fatty acids and amino acids. Anaerobic metabolism, as a main indicator of oxygen limitation was estimated by the activity of the key enzyme of the anaerobic glycolysis—lactate dehydrogenase and its end product—lactate.

Oxygen content largely differs especially in winter between the ice-covered Lake Baikal and the surrounding waters, the habitat of *G. lacustris,* which becomes supersaturated in Lake Baikal due to primary production under the ice, but possibly hypoxic in small completely frozen ponds like the sampling site of *G. lacustris*. To evaluate the ability to withstand reactive oxygen species, natural byproducts of aerobic metabolism increasingly produced both under hypoxic and hyperoxic conditions, we estimated the activity of three antioxidant enzymes. Peroxidase is responsible for the organic peroxides’ decomposition; catalase is more specific to hydrogen peroxide. Glutathione S-transferase provides the complex defense against peroxides and has a function of xenobiotic detoxication^26^.

## Results

### Energy metabolites

**Figure 2:**
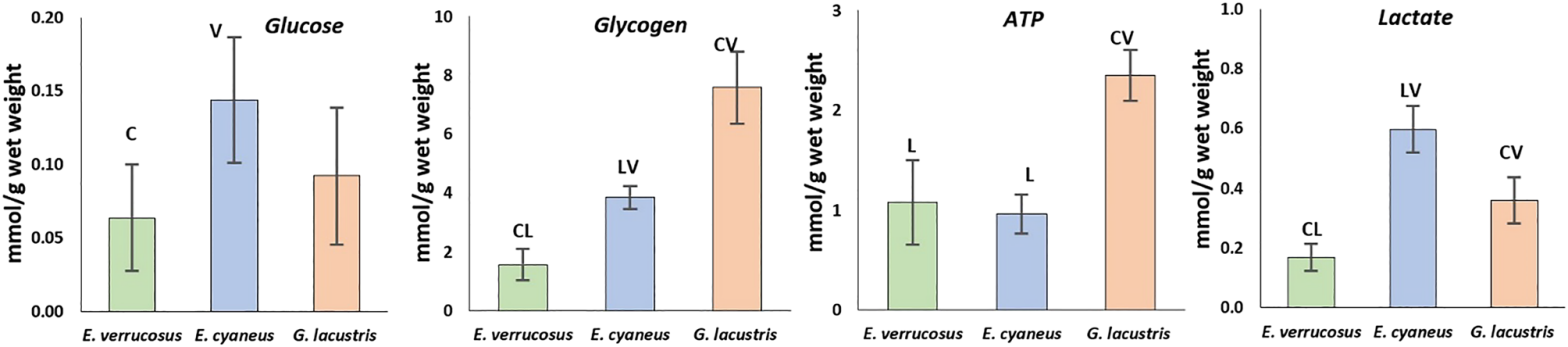
Control levels of metabolite contents in Baikal endemic amphipods *E. verrucosus* and *E. cyaneus*, as well as in Holarctic *G. lacustris* after 6 weeks of acclimation to their respective preferred temperatures. Data are presented as means ± standard deviation of the mean. C, V, L indicate *E. cyaneus*, *E. verrucosus* and *G. lacustris*, respectively, and indicate a significant difference in the activity of the markers from the bar marked with this symbol (p < 0.05).

**Figure 3:**
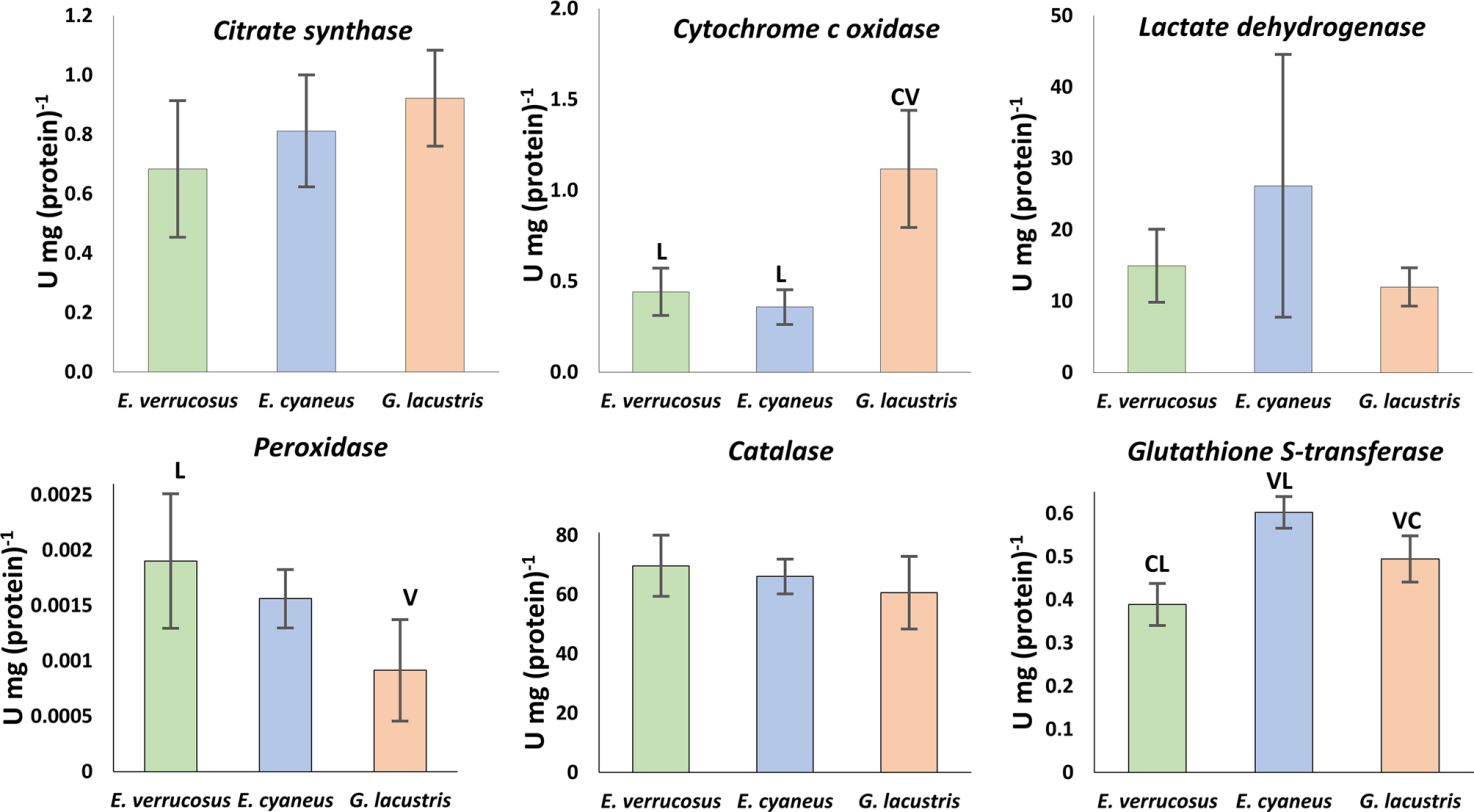
Control levels of enzyme activities in the Baikal endemic amphipods *E. verrucosus* and *E. cyaneus*, as well as in the Holarctic *G. lacustris* after 6 weeks of acclimation to their respective preferred temperatures. Data are presented as means ± standard deviation of the mean. C, V, L indicate *E. cyaneus*, *E. verrucosus* and *G. lacustris*, respectively, and indicate a significant difference in the marker’s activity from the bar marked with this symbol (p < 0.05).

**Figure 4:**
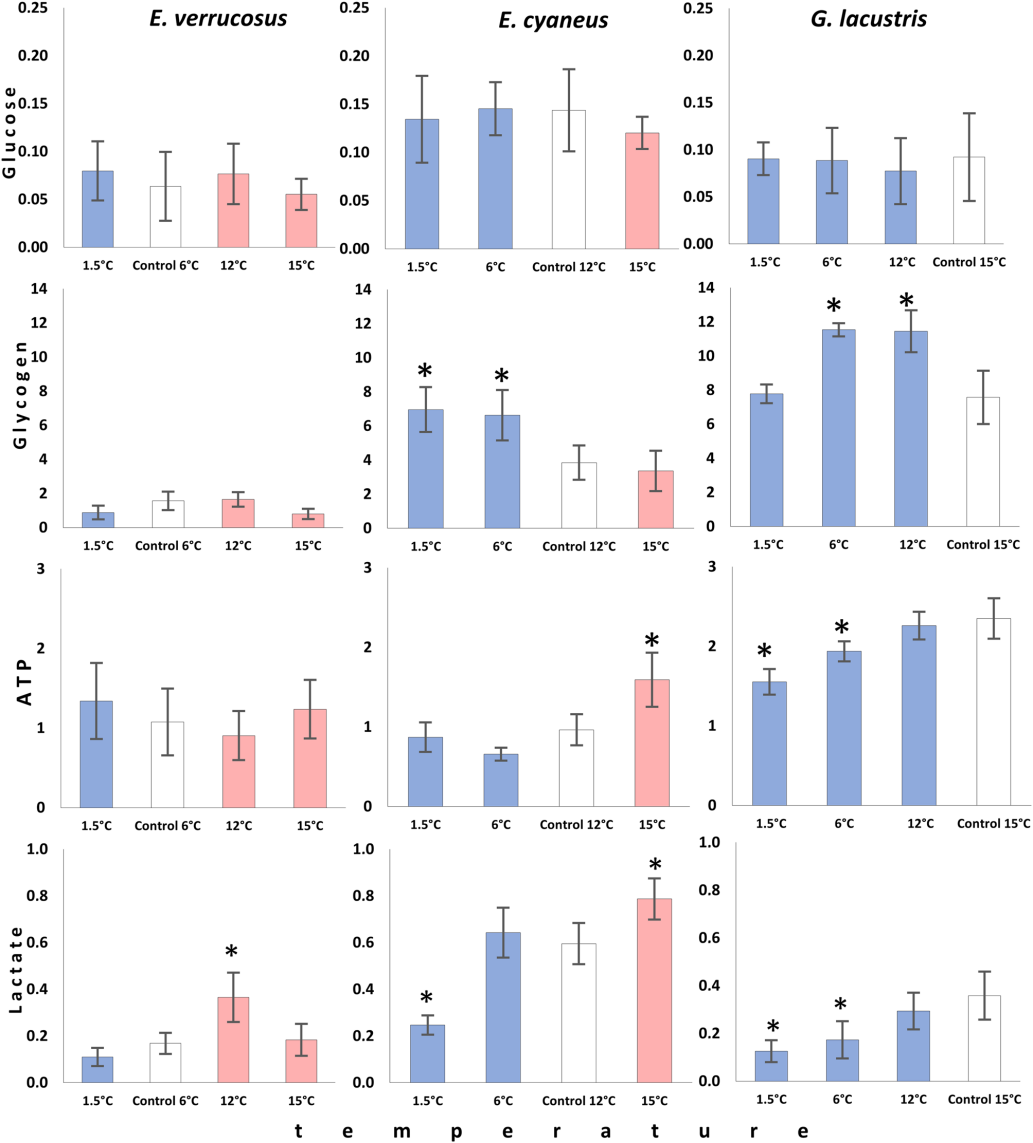
Glucose, glycogen, ATP, and lactate content (mmol/g wet weight) in *E. verrucosus*, *E. cyaneus* and *G. lacustris* after long-term exposures to temperatures different from preferred. White bars denote the preferred temperature of the species (control), while the red bars denote experimental temperatures above the preferred, and blue bars those below the preference temperatures. The data are presented as means ± standard deviation of the mean. Replicates numbers (n) for each experimental temperature were: 3–5 (9–15 individuals) for *E. verrucosus*; 4 (56 individuals) for *E. cyaneus*, and 3–4 (33–44 individuals) for *G. lacustris*. *Significant difference from initial control values (p < 0.05).

**Figure 5:**
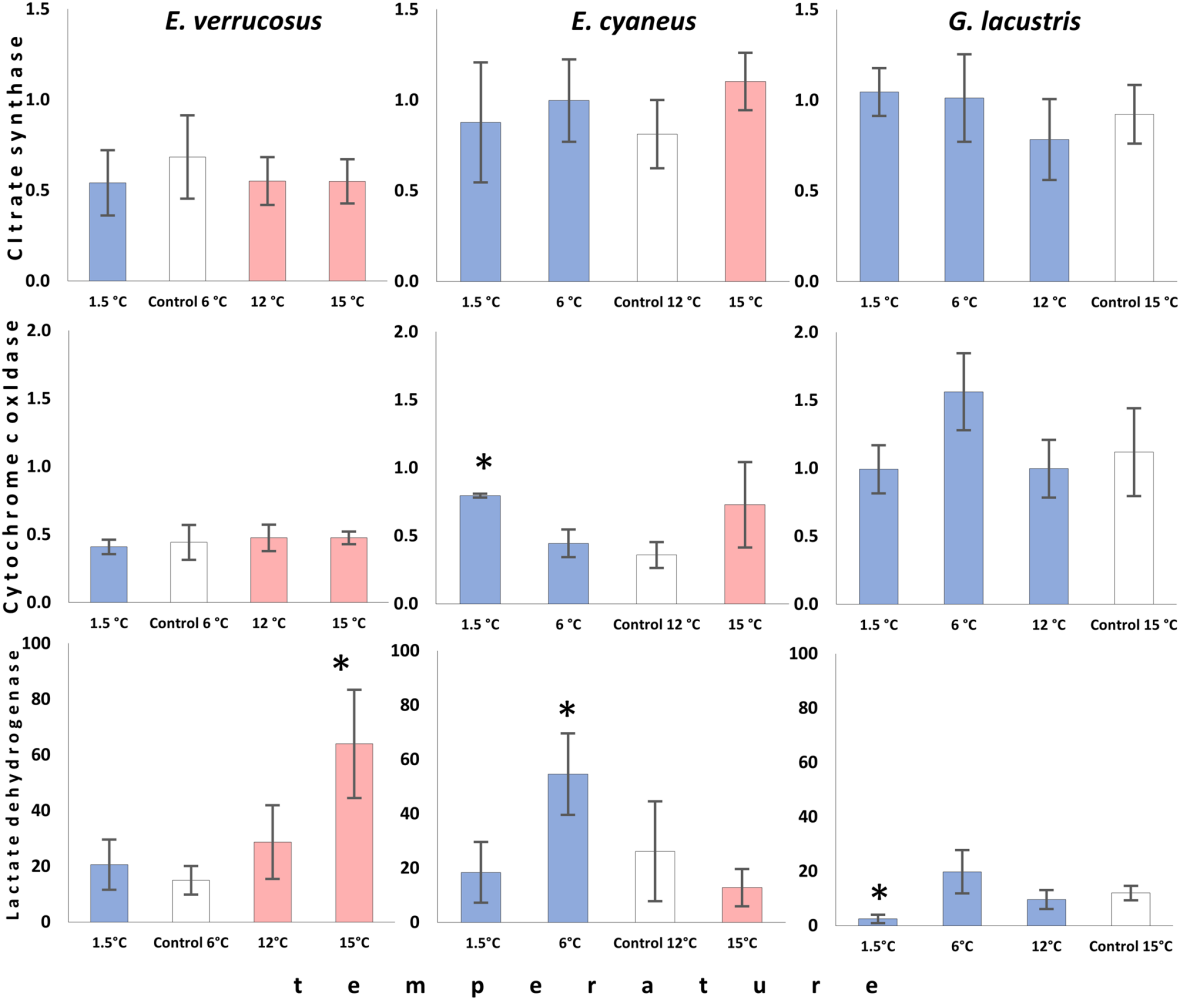
Cytochrome c oxidase, citrate synthase, and lactate dehydrogenase activity (U mg/(protein)) in *E. verrucosus*, *E. cyaneus* and *G. lacustris* after long-term acclimation to non-preferred temperatures. White bars denote the preferred temperatures of each species (control), while the red bars denote temperatures above, and blue bars those below the preferred temperatures. The data are presented as means ± standard deviation of the mean. Replicates numbers (n) for each experimental temperature were: 4–5 (12–15 individuals) for *E. verrucosus*; 4–6 (56–86 individuals) for *E. cyaneus*, and 4–6 (44–66 individuals) for *G. lacustris*. *Significant difference from control (p < 0.05).

To evaluate the effect of different temperatures on energy metabolism, the levels of glucose, glycogen, ATP, and lactate as well as the activities of key enzymes of the energy metabolism (cytochrome c oxidase, citrate synthase, and lactate dehydrogenase) were measured in the three amphipod species after acclimation to the respective preferred temperatures (control, Figs. 2, 3) and after the subsequent six weeks exposures to non-preferred temperatures (Figs. 4, 5).

Two-way ANOVA (Supplementary 1) revealed interspecific differences in glucose content (F = 18.527, p < 0.001), glycogen (F = 300.296, p < 0.001), lactate (F = 106.469, p < 0.001) and the activities of citrate synthase (F = 20.998, p < 0.001), cytochrome c oxidase (F = 68.645, p < 0.001) and lactate dehydrogenase (F = 22.605, p < 0.001). Since each species has its own control temperature, we further chose one-way-ANOVA or the Kruskal–Wallis H test (in case of not-normally distributed data) to compare the non-preferred temperature treatments with the respective preferred temperature group.

After six weeks of acclimation to the preferred temperatures, glucose levels differed significantly between the two Baikal species (F = 4.327, p = 0.048) and were about two-fold higher in *E. cyaneus* (0.14 ± 0.04 mmol/g wet weight) compared to *E. verrucosus* (0.06 ± 0.03 mmol/g wet weight) (Fig. 2). The control value of glucose in *G. lacustris* (0.09 ± 0.04 mmol/g wet weight) was intermediate and not significantly different from those of the two Baikal species. None of the species showed significant changes in glucose content during the exposures to the non-preferred temperatures (Fig. 4).

Glycogen contents differed significantly between all studied species (F = 34.853, p < 0.001), with the highest levels in *G. lacustris* (7.57 ± 1.57 mmol/g wet weight) and the lowest in *E. verrucosus* (1.40 ± 1.38 mmol/g wet weight) (Fig. 2). In *E. cyaneus*, the glycogen control value was 3.85 ± 1.00 mmol/g wet weight. In *E. verrucosus,* the glycogen content did not change in dependence of temperature. On the contrary, in *E. cyaneus*, a significant increase in glycogen content after exposure to temperatures below the preferred temperature was found (F = 8.699, p = 0.002) (1.5 °C; 6.96 ± 1.32 mmol/g wet weight and 6 °C; 6.63 ± 1.48 mmol/g wet weight). Similar, for the Holarctic species, *G. lacustris*, a significant increase in glycogen content was observed after long-term exposure to the temperatures below the preferred temperature (6 °C: 11.54 ± 0.39 mmol/g wet weight and 12 °C: 11.45 ± 1.23 mmol/g wet weight), but on the level of the control group at the coldest exposure temperature of 1.5 °C (F = 17, 0.639, p < 0.001) (Figs. 3, 4).

The control value of ATP in *G. lacustris* (2.35 ± 0.25 mmol/g wet weight) was more than two-fold higher compared to both Baikal endemics (0.96 ± 0.19 mmol/g wet weight for *E. cyaneus* and 1.07 ± 0.42 mmol/g wet weight for *E. verrucosus*) (F = 22.283, p < 0.001) (Fig. 2). In *E. verrucosus*, the ATP content did not significantly change during the different thermal exposures. In contrast, the ATP content increased in *E. cyaneus* when exposed to a temperature higher than preferred (15 °C: 1.59 ± 0.33 mmol/g wet weight) (F = 12.975, p < 0.001). For *G. lacustris*, the ATP content was significantly reduced when acclimated to low temperatures − 1.5 °C (1.55 ± 0.16 mmol/g wet weight) and 6 °C (1.94 ± 0.13 mmol/g wet weight) (F = 15.191, p < 0.001) (Fig. 4).

The lactate control level differed significantly between all studied species with about threefold higher levels in *E. cyaneus* (0.60 ± 0.09 mmol/g wet weight) compared to *E. verrucosus* (0.17 ± 0.05 mmol/g wet weight) (F = 27.534, p < 0.001). Lactate values in *G. lacustris* were intermediate (0.36 ± 0.10 mmol/g wet weight) (Fig. 2). Long-term exposure of *E. verrucosus* to 12 °C (higher than the preferred temperature) led to significantly increased lactate levels compared to the preferred temperature of 6 °C (0.37 ± 0.11 mmol/g wet weight) (F = 10.049, p = 0.001). At 15 °C, the lactate content turned back to the control level. For *E. cyaneus*, a decrease in lactate content was observed at 1.5 °C (0.25 ± 0.04 mmol/g wet weight) (F = 29.297, p < 0.001) compared to the preferred temperature of 12 °C, but higher lactate content was found after warming (15 °C: 0.79 ± 0.09 mmol/g wet weight) (F = 29.297, p < 0.001). In *G. lacustris*, the levels of lactate were significantly lower after the exposure to colder than the preferred temperatures, both at 1.5 °C (0.12 ± 0.04 mmol/g wet weight) and at 6 °C (0.17 ± 0.08 mmol/g wet weight) (F = 7.550, p = 0.004) (Fig. 4).

### Maximum capacities of key metabolic enzymes

Enzyme capacities for cytochrome c oxidase measured at the preferred temperature of 15 °C were 2–3 times higher in the Holarctic *G. lacustris* (1.12 ± 0.32 U/mg/(protein)) compared to both Baikal endemics (*E. verrucosus* (0.44 ± 0.13 U/mg/(protein)) and *E. cyaneus* (0.36 ± 0.10 U mg/(protein))) (F = 16.336, p = 0.001) (Fig. 2). During the experimental exposure, only the more eurythermal Baikal endemic *E. cyaneus* doubled its enzyme capacity when acclimated below its preference temperature 0.79 ± 0.01 U mg/(protein)) at 1.5 °C relative to 0.36 ± 0.09 U mg/(protein)) at 12 °C (Kruskal–Wallis H test, H = 7.831, p = 0.050). For *G. lacustris* and *E. verrucosus*, cytochrome c oxidase activity remained unchanged (Fig. 5). Instead, the key enzyme of the central metabolic hub in mitochondria, the citrate synthase, neither differed between the three studied species nor responded significantly to any temperature treatment besides some reasonable variation.

Relatively high lactate dehydrogenase activities were determined in *E. cyaneus* at the control temperature compared to the other two species but differences remained statistically insignificant likely due to the high inter-individual variation in *E. cyaneus* (Fig. 3). A statistically significant fourfold increase in lactate dehydrogenase activity (64 ± 19.4 U/mg protein) relative to the control (6 °C: 20.6 ± 9 U/mg protein) was found in *E. verrucosus* when warm-acclimated to 15 °C (F = 13.844, p < 0.001). Instead, cold acclimation of *E. cyaneus* to 6 °C led to a two-fold increase in lactate dehydrogenase activity (54.6 ± 15 U/mg protein) relative to the control level (12 °C: 26.2 ± 18.4 U/mg protein: F = 8.408, p = 0.001). At 1.5 °C, such a response was not found. Besides the low control levels in *G. lacustris*, a nearly fivefold decrease in lactate dehydrogenase activity was shown during long-term cold exposure at 1.5 °C (2.5 ± 1.5 U/mg protein) relative to the preference temperature (15 °C: 12 ± 2.7 U/mg protein: Kruskal–Wallis H test, H = 13.905, p = 0.003) (Fig. 5).

### Maximum capacities of antioxidant enzymes

**Figure 6:**
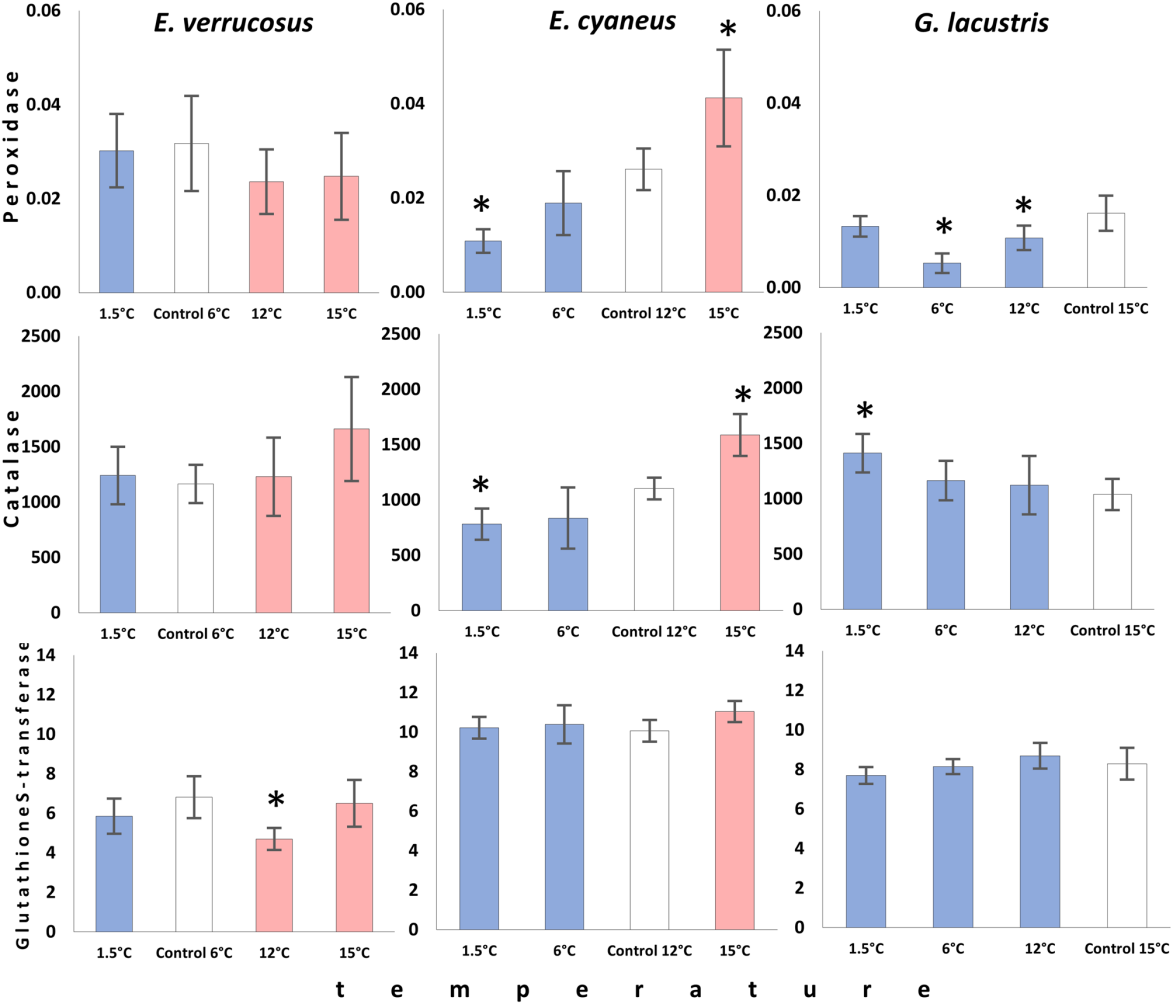
Peroxidase, catalase, and glutathione S-transferase activity (nkat/mg protein) in *E. verrucosus*, *E. cyaneus*, and *G. lacustris* after long-term acclimation at temperatures different from each species’ preferred temperature. White bars denote the preferred temperature of each species (control), while the red bars denote temperatures above the preferred, and blue bars those below the preferred temperatures. The data are presented as means ± standard deviation of the mean. Replicates numbers (n) for each experimental temperature were: 4–6 (12–18 individuals) for *E. verrucosus*; 5–6 (70–84 individuals) for *E. cyaneus*, and 4–6 (44–66 individuals) for *G. lacustris*. *Significant difference from control (p < 0.05).

Antioxidative enzyme activities differed between the studied amphipods when exposed to the respective preference temperatures and responded species-specifically to temperature as demonstrated by both two-way (S1) and one-way ANOVA. Control levels of maximum peroxidase activities were two-fold lower in *G. lacustris* (0.016 ± 0.003 nkat/mg protein (0.000.915 ± 0.000.458 U/mg protein)) compared to *E. verrucosus* (0.032 ± 0.010 nkat/mg protein (0.00.190 ± 0.000.607 U/mg protein)), whereas *E. cyaneus* showed intermediate activities (0.026 ± 0.004 nkat/mg protein (0.00.156 ± 0.000.264 U/mg protein)) (F = 6.483, p = 0.011) (Fig. 3). In *E. verrucosus*, temperature did not significantly affect peroxidase activity (Fig. 6). Instead, peroxidase activity was decreased in *E. cyaneus* individuals acclimated to a temperature below the preferred—1.5 °C (0.011 ± 0.002 nkat/mg protein) relative to the control (0.026 ± 0.004 nkat/mg protein) (F = 21.287, p < 0.001). On the warm side at 15 °C, a significant increase in peroxidase activity was found in *E. cyaneus* (0.041 ± 0.010 nkat/mg protein) (F = 21.287, p < 0.001). Similar, long-term cold exposure of *G. lacustris* resulted in a significant decrease in peroxidase activity, both at 12 °C (0.011 ± 0.003 nkat/mg protein) and at 6 °C (0.005 ± 0.002 nkat/mg protein) relative to the control at 15 °C (0.016 ± 0.004 nkat/mg protein) (F = 11.180, p < 0.001). This trend was reversed at the lowest exposure temperature of 1.5 °C, with capacities comparable to control levels (Fig. 6).

Catalase capacities were comparable in all amphipods at their preferred temperature (Fig. 3). As for peroxidase, long-term thermal exposures of *E. verrucosus* did not lead to significant changes in catalase activity (Fig. 6). Again, a unidirectional downward trend in enzyme capacities with lower temperature became evident for *E. cyaneus,* as catalase activities decreased significantly at 1.5 °C (782 ± 141 nkat/mg protein) relative to the control individuals at 12 °C (1104 ± 99 nkat/mg protein) (F = 21.408, p < 0.001). Furthermore, catalase activity was higher on the warm side (15 °C: 1587 ± 189 nkat/mg protein) than at the control temperature. In contrast, catalase capacities in *G. lacustris* increased slightly by about 40% at 1.5 °C (1412 ± 173 nkat/mg protein) relative to the animals kept at the preference temperature (15 °C: 1039 ± 141 nkat/mg protein) (F = 3.894, p = 0.026) but remained unchanged at the intermediate temperature treatments.

Glutathione S-transferase activity differed significantly between all species and was highest in *E. cyaneus* (10.06 ± 0.55 nkat/mg protein (0.60 ± 0.04 U/mg protein)) followed by *G. lacustris* (8.29 ± 0.80 nkat/mg protein (0.49 ± 0.05 U/mg protein)) and *E. verrrucosus* (6.82 ± 1.06 nkat/mg protein (0.39 ± 0.05 U/mg protein)) (F = 22.920, p < 0.001) (Fig. 3). None of the long-term thermal exposures affected glutathione S-transferase activities in *G. lacustris* and *E. cyaneus*. In *E. verrucosus*, a 30% reduction of glutathione S-transferase capacities became significant (F = 5.801, p = 0.005) at 12 °C (4.69 ± 0.55 nkat/mg protein) relative to the preference temperature (6 °C: 6.82 ± 1.06 nkat/mg protein), but at 15 °C, control levels were found again.

## Discussion

This study aimed to investigate species-specific thermal adaptations of two endemic amphipod species differing in their thermal tolerance and one widespread Holarctic species by comparing long-term responses to cold and warm temperatures beyond their respective preference temperatures. We hypothesize that the Holarctic *G. lacustris* is limited at the extreme low temperature (i.e. 1.5 °C) preventing this species from establishing a stable population in Lake Baikal, whereas the Baikal endemic amphipods possessing specific thermal adaptations to maintain a high energy metabolism in winter at low temperatures.

To test this hypothesis, we measured metabolic markers in long-term acclimated animals exposed to four different temperatures (i.e., 1.5 °C, 6 °C, 12 °C and 15 °C) including the respective preference temperature of each species. More specifically , we studied key metabolic enzyme activities, such as citrate synthase and cytochrome c and oxidase, to estimate aerobic energy production and lactate dehydrogenase as an indicator of anaerobic glycolysis. To better understand the metabolic output of these enzymes we studied metabolite contents such as ATP, lactate, and glycogen. Finally, we investigated activities of three antioxidant enzymes to evaluate the level of cellular stress. Overall, we could demonstrate significant differences of several markers between all three species and their responsiveness to temperature. Common patterns and their implications for the performance of the species in their habitats are discussed in the following.

### Metabolic properties at optimal temperatures

Long-term acclimation to the preferred (= optimal) temperatures allows maintaining homeostasis and avoids any stress related to the non-optimal thermal conditions. At these optimal temperatures, the aerobic scopes of the species should be at their maxima meaning that maximum excess oxygen is available to fuel processes above maintenance to support e.g., growth and reproduction^7,27^.

Here, we found differences in the capacities of aerobic metabolism, levels of energy equivalents and energy stores between the three species, as indicated by the higher activity of cytochrome c oxidase, ATP and glycogen levels in *G. lacustris* compared to the two Baikal species. This is in line with our previous findings about routine metabolic rates, measured as oxygen consumption in the studied species^21^.

Constantly low temperatures led to decreased cellular ATP levels and activities of cytochrome c oxidase in Baikal endemics, thereby indicating a reduced rate of basal metabolism. The same trend was observed in Antarctic fish compared to temperate and tropical fish species^27,28^.

However, the activity of another marker of aerobic metabolism—citrate synthase—is similar among the three species. The higher citrate synthase/cytochrome c oxidase ratio in Baikal species compared to *G. lacustris* may be attributed to the prevalence of lipid over glucose metabolism in Baikal species^29^. Significantly higher levels of glycogen in *G. lacustris* support this assumption.

Here, we studied one thermally tolerant Baikal endemic species, *E. cyaneus*, and one cold-stenothermal species, *E. verrucosus*. Previous studies on the molecular evolution of thermal tolerance revealed significant structural and functional differences in heat shock proteins (Hsp70), a universal molecular protection system^24^. Significant differences in routine metabolic rate and lethal temperatures were also shown^21^, supporting both endemic species’ thermal classification. The present results widen our mechanistic understanding of thermotolerance in Baikal amphipods. Particularly, when acclimated to their preferred temperatures, the two Baikal species showed some differences in metabolic fuel usage. Higher glucose, glycogen and lactate levels correspond to a more enhanced glucose metabolism, which may be required to maintain higher metabolic activity of the thermotolerant species *E. cyaneus.* However, capacities of cytochrome c oxidase and citrate synthase activities are similar, as well as the ATP level, which indicates similar rates of oxygen metabolism under optimal thermal condition in these two species.

Oxygen metabolism is tightly connected with the antioxidative defense against reactive oxygen species (ROS) as they are natural byproducts of aerobic metabolism. Therefore, we studied the activity of three antioxidative enzymes. Our results indicate that despite higher aerobic capacities and presumably higher metabolic rates in *G. lacustris* compared to the two Baikal species, the activities of catalase and glutathione S-transferase were comparable and for peroxidase even higher in the cold stenothermal *E. verrucosus* than in *G. lacustris* (Fig. 2). Similarly, Abele and Puntarulo^24,30^ showed that the basic levels of superoxide dismutase activity in polar mollusks are higher than in temperate ones. Increased ROS generation in cells of ectothermic animals may occur due to higher oxygen solubility in cold water and biological fluids^31,32^. Thus, higher peroxidase level in *E. verrucosus* is likely an adaptation to the low temperatures with its high dissolved oxygen content in Baikal water.

Glutathione S-transferase activities were found to be higher in both thermotolerant species—*G. lacustris* and *E. cyaneus*. Besides protecting against ROS, this enzyme has multiple functions, including xenobiotic detoxification^33^. Thus, its higher activity may be related to the enhanced tolerance level to various environmental factors in both species besides temperature.

### Thermal exposure to lower than the preferred temperatures

We hypothesized that Baikal species have metabolic adaptations to cold temperatures that allow an active lifestyle in the cold. Thus, we expected cold compensation mechanisms (i.e., increased enzyme activities due to higher densities of mitochondria in cold stenotherms). Our results indicate that this is the case only for *E. cyaneus* (Baikal endemic, thermotolerant), as it showed increased cytochrome c oxidase activities at 1.5 °C. As the citrate synthase activity and ATP level remained unchanged, compensation of the respiratory chain seems to be sufficient to support stable energy production at low temperatures and an alteration of the mitochondrial ultrastructure, i.e., increased cristae density (cytochrome c oxidase) relative to the matrix (citrate synthase) may be involved in cold acclimation.

*E. cyaneus* represents the rather small summer-reproducing complex among Baikal endemic amphipods. Despite its relatively high thermotolerance^24^, this species is actively moving under the ice cover in winter. In our experiments, animals of this species expressed high locomotor and feeding activity at all exposure temperatures, including 1.5 °C. Our results unravel the metabolic adaptation providing this rather wide thermotolerance window of this species. Already at 6 °C, glycogen started to accumulate, which also reoccurred at 1.5 °C. Glycogen accumulation is a well-known strategy of overwintering ectotherms known as cold hardiness, especially in those surviving in frozen habitats under oxygen-limited conditions^34^. Similar, we found an accumulation of glycogen above the control level in the Holarctic *G. lacustris* when exposed to 12 and 6 °C. However, at 1.5 °C the level of glycogen in this species was below the control level, indicating reduced glycogenesis possibly due to metabolic depression in the cold.

Instead, *E. cyaneus* can maintain a high level of glycogenesis at 1.5 °C, and therefore accumulate glycogen in winter, despite its lower metabolic rate compared to *G. lacustris*. Surprisingly, at 6 °C we found a strong increase in lactate dehydrogenase activity in *E. cyaneus*, which was not followed by a significant accumulation of lactate or significant depletion of ATP indicating the absence of functional anaerobiosis. This unpredicted reaction of lactate dehydrogenase in this species at 6 °C, which is the Baikal littoral zone’s annual average temperature, requires further studies.

Exposure to 1.5 °C and 6 °C caused decreased catalase and peroxidase activities and decreased lactate levels in *E. cyaneus*, which would be in line with a reduced metabolic rate, following the Q10 rule. Thus, our results indicate that *E. cyaneus* can maintain a high level of aerobic metabolism within a wide thermal range including the common winter temperature—1.5 °C. Although *E. cyaneus* is one of the most thermotolerant Baikal amphipod species, differences to the closely-related thermotolerant Holarctic *G. lacustris* became apparent as the latter failed to maintain high ATP and lactate levels at the lowest exposure temperature likely due to lacking compensation (citrate synthase, cytochrome c oxidase) or even inactivation of enzyme functions (including lactate dehydrogenase). This reduced energy state may likely contribute to the low locomotor activity of *G. lacustris* at the lowest exposure temperature.

Surprisingly, no cold compensation in any of the studied parameters was found for the cold-loving Baikal endemic species *E. verrucosus*. This species belongs to the winter-reproducing complex, which is most common in the littoral zone. The absence in cold compensation of this species indicates that at 1.5 °C its energy metabolism remains on its physiological maximum.

The absence of glycogen accumulation at low temperatures in this species can be explained by the food availability during winter in Lake Baikal’s littoral zone. Presumably, the existing amount of winter nutrition is sufficient for *E. verrucosus* to maintain maximum aerobic capacities and energy metabolism. The high nutrition allows the species to breed, develop eggs and release juveniles, which occurs during the seasons with lowest annual temperatures. Another explanation can be the usage of lipids instead of carbohydrates as energy source in winter. The lower amount of glycogen and free glucose than in the thermotolerant congener *E. cyaneus*, supports this hypothesis.

Thus, despite the relatively similar aerobic capacities of the thermotolerant *E. cyaneus* and the winter-reproducing cold stenothermal *E. verrucosus* at their respective preference temperatures, the latter species shows a different metabolic fuel use, that allows *E. verrucosus* to maintain its maximum metabolic rate at 1.5 °C.

We hypothesized that the Holarctic *G. lacustris* has disadvantages compared to the two Baikal species regarding energy metabolism at low temperatures. *Gammarus lacustris* is eurythermal and often overwinters in small ponds, which are nearly completely frozen in winter^35^. However, it has been shown that temperatures below its optimal range cause decreased respiration rates and activity possibly resulting in torpor^21,35^. Our observations confirmed these assumptions as at 1.5 °C individuals of *G. lacustris* significantly decreased their locomotor and feeding activities. The assumption that *G. lacustris* shows metabolic depression at 6 °C and 1.5 °C is supported by the decreased ATP, lactate, and lactate dehydrogenase activity levels. Moreover, only for this species we observed mortality at 1.5 °C, which indicates that this experimental thermal condition is stressful. This is supported by the increase in catalase activity over the control level and the elevated level of peroxidase activity at 1.5 °C, while at 12 °C and 6 °C, the capacity for this enzyme was decreased compared to the control (following the Q10 rule). In this case, exposure to 1.5 °C could cause cellular damages resulting in the development of oxidative stress. Preparation for such extreme temperatures for *G. lacustris* like requires enough time to accumulate cryoprotectors^25^ and adjust the metabolism in its natural habitat. Besides, we observed the accumulation of glycogen, like in the thermotolerant *E. cyaneus*. For *G. lacustris*, accumulation of glycogen for the wintertime is essential, as it is often overwintering in nearly completely frozen ponds and therefore experiences periods of hypoxia^23^. Glycogen can be metabolized via anaerobic glycolysis, in contrast to lipid storages, which require oxygen for their metabolizations^34^. In comparison to *E. cyaneus*, accumulation of glycogen over the control level in *G. lacustris* occurs already at 12 °C, indicating the lower thermal threshold of the zone of preferred temperatures for this species^22^, but this accumulation disappeared at the lowest temperature indicating that *G. lacustris* follows a different strategy for winter survival than cold hardiness^34^.

### Thermal exposure to higher than the preferred temperatures

Previous studies indicated that 15 °C is the critical thermal threshold for large adults of the stenothermal *E. verrucosus,* and it was observed that most of these individuals start to migrate to deeper littoral zones when the temperature in the Baikal littoral surpass about 11 °C^21^. Thus, *E. verrucosus* is behaviorally adapted to escape deleterious temperatures. When exposed to gradual temperature increase, individuals of *E. verrucosus* showed accumulation of lactate and heat shock proteins at temperatures exceeding 12 °C^22,24^. Our results complement these previous findings, as lactate levels were significantly higher than the control lactate levels at 12 °C. At 15 °C, increases of lactate dehydrogenase capacities were detected, which may foster a higher turnover and remobilization of lactate at this temperature compared to 12 °C, as lactate level did not further increase. As shown earlier lactate dehydrogenase was the only metabolic enzyme exhibited similar kinetic and regulatory properties as the other two amphipod species (following simple Q10 rules)^23^. The results here confirm the need of *E. verrucosus* to keep higher anaerobic capacities and turnover rates at the upper limit of the thermal window, where functional hypoxia may appear^7,27^.

In the more thermotolerant *E. cyaneus*, exposure to 15 °C caused increases of ATP, lactate, and both peroxidase and catalase activities. Increases of both ATP and lactate likely indicate the enhancement of the cellular respiration rather than the onset of anaerobiosis. Activation of peroxidase and catalase may indicate both the development of cellular stress and the general increase in metabolic rate following the Q10 rule. The last explanation is more likely, as antioxidant enzyme activities, lactate and ATP content gradually increased with rising temperature. As *E. cyaneus* is a sedentary species, that occupies a rather narrow zone of the upper littoral, such a metabolic plasticity can serve as a specific adaptation to its thermal niche. Opposite, for the migrating *E. verrucosus*, behavioral responses are more important when temperatures gradually increase.

## Conclusions

This study sheds light on the metabolic adaptations of Baikal endemic amphipods to chronic low temperatures. Winter in Lake Baikal provides a unique opportunity for its endemics—a high level of dissolved oxygen and a winter/spring under-ice algae bloom providing food in the otherwise highly oligotrophic ecosystem. While cold stenothermal species have a clear benefit in winter conditions, as they are adapted to reproduce under ice, eurythermal species developed the abilities to keep high metabolic activities at low temperatures without performing their reproduction cycle, in contrast to their congeners from the adjacent waterbodies slowing down their metabolism.

Here we detected species-specific metabolic adjustments of Baikal stenothermal species (representing the upper-middle macrozoobenthic community of the lake) to low temperatures. Overall low metabolic rates and limited responsiveness seem to be the main metabolic reaction norm. This allows keeping maximum metabolic capacity both at the optimal temperature and at 1.5 °C. At the same time, the activity of peroxidase in *E. verrucosus* is enhanced, compared to the two other studied species, which indicates activity of the cellular defense system against ROS in the oxygen-rich environment. Compared to the eurythermal endemic species (*E. cyaneus*), different use of energy fuels with preferred usage of lipids instead of carbohydrates as energy source in winter is another important metabolic adaptation to cold temperatures.

Both Baikal endemics showed lower aerobic metabolic capacities but higher anaerobic capacities at all temperatures compared to *G. lacustris*. This is somehow surprising, since the Lake Baikal is not only in winter, but throughout the year more oxygen-rich compared to the surrounding water, the habitat of *G. lacustris*. As the highest temperature used in this experimental setup represented the preference temperature of *G. lacustris*, any hypoxic stress due to functional hypoxia can be excluded and higher anaerobic capacities will only evolve at habitat-specific maximal temperatures (beyond 20 °C). Besides these higher aerobic capacities, *G. lacustris* failed to maintain stable ATP levels at the lowest temperature, indicating severe limitations of its metabolism that prevent an active mode of life. Although its higher potential at warm temperatures may be beneficial under future warming conditions^21,23^, this study corroborates on a cell physiological basis that different mechanisms to cope with cold temperatures are a key component of the species immiscibility barrier, preventing *G. lacustris* from establishing a stable population among the indigenous fauna of Lake Baikal.

## Material and methods

### Sampling and animal maintenance

Sampling was carried out in 2017 from August 8th until August 18^th^ for the Baikal species and until August 26th—for *G. lacustris*. The two Baikal species, *E. cyaneus* and *E. verrucosus,* were caught with a hand net in Lake Baikal’s littoral at depths of 0.3–1 m near the village of Bolshie Koty (51° 54′ 11.67″ N, 105° 4′ 7.61″ E). This sampling site is a rocky beach covered with algae and characterized by intense hydrodynamics (wave movements). The Holarctic species *G. lacustris* was caught in “Lake 14” close to the same village (51° 55′ 14.39″ N, 105°4′ 19.48″ E), which is a small eutrophic freshwater pond of artificial origin (rest of gold mining activities) located about 2 km from Lake Baikal. This reservoir is inhabited by species that are widely distributed in the Palearctic and Holarctic.

During sampling, amphipods were sorted by species and kept in aerated tanks at the sampling temperature (7 ± 0.5 °C for the two Baikal species and 13 ± 0.5 °C for the Holarctic species). During acclimation, Baikal endemic amphipods were fed by a custom-made feeding mixture including dried and ground amphipods, algae, aquatic plants, and detritus collected in the sampling area. *Gammarus lacustris* was fed with wet leaves and detritus collected from its sampling site, and commercial dried *Gammarus* sp. (presumably *G. lacustris*; Barrom, Barnaul, Russia) from Siberian lakes. Commercial dried Gammarus was used because the Holarctic species *G. lacustris* is prone to cannibalism. Amphipods were kept in dim light to mimic the natural environment. The animals were placed in 2-L aquaria (n = 3 for each temperature), each containing 200 individuals of *E. cyaneus*, 75 individuals of *G. lacustris* and, 30 individuals of *E. verrucosus*, respectively.

For the Baikal species, water from Lake Baikal was used, and for the Holarctic species, filtered water from its habitat. The water was changed at least once every three days. High locomotor and feeding activity during the acclimation period indicated that the amphipods’ maintenance in the laboratory was not stressful. Only actively swimming adult individuals were used for the experiments.

### Experimental setup

The acclimation temperature was gradually adjusted to the respective preference temperatures, as shown in our previous research—6 °C for *E. verrucosus*, 12 °C for *E. cyaneus*, and 15 °C for *G. lacustris*^6,22^. The temperature change rate was 1 °C per day in case of temperature decrease and 1.5 °C per day in case of temperature increase. Amphipods were then pre-acclimated at the preferred temperatures for two weeks (to ensure recovery after sampling and transportation) before the main part of the experiment. The preferred temperatures were chosen as controls for each species as they correlate with the most stable values of biochemical stress markers^22^.

After pre-acclimation, amphipods of each species were divided into four equal groups for temperature exposures. Each group consisted of 30 specimens of *E. verrucosus*, 200 sp. of *E. cyaneus* and 75 sp. of *G. lacustris* kept in 2 L aquaria (n = 3). Different numbers of exposed specimens were due to the different sizes of animals.

The four thermal treatments included: (1) species-specific preferred temperature, (2) hypothermic exposure (1.5 °C), (3 and 4) preferred temperatures of the other two species (6, 12, and 15 °C, respectively).

To achieve the target temperatures after pre-acclimation, the temperature was gradually decreased/increased at a rate of 1–1.5 °C per day to 1.5, 6, 12, and 15 °C, respectively. Subsequently, amphipods were kept at the respective temperatures for one month. Water (Baikal water for Baikal species and filtered water from *G. lacustris* habitat) was exchanged every third day and amphipods were fed as during preacclimation.

At the end of the experimental exposure, the animals were flash-frozen in liquid nitrogen for further biochemical analysis. The number of biological replicates was 6–10. Experimental temperatures were maintained by an incubator (Sanyo MIR-254 (238l), Osaka, Japan). The temperature was gradually increased/decreased using a thermostat (WiseCircu, Witeg GmbH, Wertheim, Germany).

### Mortality and behavior

During long-term thermal exposure of Baikal and Holarctic amphipods, some mortality occurred in both Baikal and Holarctic species (Table 1).

**Table 1:** Mortality and cannibalism rates of Baikal endemic amphipod species *E. verrucosus, E. cyaneus*, and the ubiquitous Holarctic species *G. lacustris* during sampling, pre-acclimation, and long-term acclimation to temperatures different from the respective preference temperature.

Temperature/species (%)	*E. verrucosus* (%)	*E. cyaneus* (%)	*G. lacustris* (%)
Sampling and pre-acclimation	6.3	1.9	***38.4***
**Long-term acclimation (1 month)**			
15 °C	6.3	0.4	***4.9***
12 °C	0.3	–	***2.9***
6 °C	–	–	***1.5***
1.5 °C	–	–	0.8

Mortalities due to cannibalism are shown in bold italics.

At temperatures above preferred (15 °C), 6.3% of the *E. verrucosus* individuals died during one month of exposure, while at 12 °C only 0.3% died. At 6 °C (the preferred temperature of this species) and 1.5 °C, none of the animals died. Both at the preferred temperature of 6 °C and the temperature below preferred (1.5 °C), *E. verrucosus* showed high locomotor and feeding activity. During the two-week sampling, and the 2-weeks laboratory pre-acclimation prior the main exposure, the mortality rate of *E. verrucosus* was 6.3% due to the possible presence of injured animals (without visible signs).

A different pattern was observed for the other Baikal species *E. cyaneus*. During the exposure to 15 °C (above preferred), the mortality rate was only 0.4%. No dead amphipods were found at 12 °C (preference temperature), 6 and 1.5 °C (temperatures below preferred). During sampling and laboratory pre-acclimation, the mortality rate of *E. cyaneus* was ≤ 2%.

For the Holarctic *G. lacustris*, it was more challenging to monitor mortality since this species is cannibalistic^36,37^. Initially, we noted that the number of animals decreased, while no dead individuals were observed. Further, cases of feeding on alive individuals (weakened or after molting) were recorded. The most significant loss due to cannibalism—38.4%—was observed during the two weeks of sampling and laboratory pre-acclimation. Therefore, the diet of *G. lacustris* thenceforth included dried Gammarus (commercial fish food), consisting of *Gammarus* sp*.* (presumably, *G. lacustris*). During acclimation to 15 °C (the preference temperature) for one month, a significant decline in losses by cannibalism was observed, from 38.4 to 4.9%. At 12 °C, the number of eaten amphipods was significantly lower (≤ 3%). At 6 °C (temperature below preferred), there were 1.5% cannibalized individuals. During long-term exposure to the lowest experimental temperature—1.5 °C, there was no longer cannibalism, but a natural mortality rate of 0.8%. To determine the percentage of cannibalism and mortality, amphipods were counted every third day.

### Biochemical methods

Deep frozen individuals of *G. lacustris* (10–12 specimens), *E. cyaneus* (12–15 sp.), and *E. verrucosus* (3 sp.) were ground to coarse powder in liquid nitrogen. The powder was aliquoted in order to measure all biochemical parameters in the same pooled sample. The different individual numbers for one pooled sample were dictated by the individual size and weight of the studied amphipod species. Test tubes contained 100 ± 30 mg for measuring cytochrome c oxidase and citrate synthase activity; 60 ± 30 mg for determining energy metabolites content (glucose, glycogen, and ATP (adenosine triphosphoric acid)); 100 ± 50 mg for determining antioxidant enzymes (peroxidase, catalase, and glutathione S-transferase) and lactate dehydrogenase activity, and 200 ± 30 mg for determination of lactate content, respectively.

### Energy metabolite isolation

To determine the content of energy metabolites (lactate, glucose, glycogen, and ATP), a solution of 0.6 M HClO_4_ with 15 mM Na-EDTA was added to a small amount of sample powder at the ratio of 1500 µl HClO_4_-Na-EDTA per 100 mg of fresh weight and further ground to a uniform homogenate. The resulting acid-soluble fraction was centrifuged at 10,000*g* for 30 min at 4 °C. The supernatant was carefully collected and neutralized with 5 M K_2_CO_3_ at a rate of 70 µl per 1 ml of the supernatant (pH 7.5). The neutralized extract was incubated in the cold for 60 min to precipitate perchlorates and then centrifuged at 10,000*g* for 15 min at 4 °C. Aliquots of the resulting supernatant were stored in liquid nitrogen until the content of energy metabolites in the samples was determined.

### Lactate content

Lactate levels were determined using the express-kit “Lactate-vital” (Vital-Diagnostics, St. Petersburg, Russia) according to modified method of Bergmeyer^38^. Absorbance was measured using a Carry 50 Conc UV/visible spectrophotometer (Varian, USA) at λ = 505 nm.

### Glucose, glycogen, and ATP content

The levels of glucose, glycogen, and ATP were measured using the NADH/NADPH-dependent enzymatic methods at λ = 340 nm according to Grieshaber et al.^39^, Bergmeyer^38^ and Sokolova et al.^8^, respectively. Glycogen was hydrolyzed using the method of Sokolova et al.^8^. Absorbance was measured with a Carry 50 Conc UV/visible spectrophotometer (Varian, USA).

### Activities of cytochrome c oxidase and citrate synthase

Fifty to hundred micrograms of sample powder were homogenized in pre-cooled 20 mM Tris HCl buffer, pH 8.0, containing 1 mM sodium ethylenediaminetetraacetate (Na-EDTA), 0.1% Triton X-100, 100 mM NaCl, a cocktail of p2714 protease inhibitors (Sigma-Aldrich, Steinheim, Germany) using a Heidolph Brinkmann Tool 18F for Silent Crusher M (Fisher Scientific, Germany) at 16,500*g*. Extraction buffer was added to the sample in a ratio of 10 µl per 1 mg. The sample was homogenized three times for 10 s, with ten-second stops for cooling. Then the tubes with homogenate were centrifuged (1000*g*) for 10 min at 4 °C. The non-purified supernatant obtained during isolation was used to measure enzyme activity using a Specord S600 spectrophotometer (Analytic Jena, Jena, Germany) according to Moyes et al.^39,40^ (cytochrome oxidase) and Sidell et al.^40,41^ (citrate synthase), with modifications by Jakob et al.^23^. The activity of each sample was measured in two dilutions (for example, 2 and 4 µl) at 15 °C. The reaction was initiated by adding substrates (cytochrome C for cytochrome c oxidase and oxaloacetate for citrate synthase).

### Activities of antioxidant enzymes

Activities of total cellular peroxidases, catalase, and glutathione S-transferase were measured using standard spectrophotometric assays. Enzyme extraction was done as described in Bedulina et al.^42^ using a 1:3 (w:v) ratio of homogenization medium to amphipod biomass. Enzyme activities were measured at 25 °C in the supernatant using a Carry 50 Conc UV/visible spectrophotometer (Varian, USA). The total peroxidase activity in the soluble fraction was measured with 4.4 mM guaiacol as a substrate at 436 nm, pH 5.0, according to Pütter ^43^. Catalase activity was measured with 2.25 mM hydrogen peroxide as a substrate at 240 nm, pH 7.0, according to Aebi^44^. Glutathione S-transferase activity was measured with 0.97 mM 1-chloro-2.4-dinitrobenzene as a substrate at 340 nm, pH 6.5^45^. The Bradford assay was used to evaluate protein concentrations^46^.

### Data analysis and statistics

Significant changes in biochemical markers between species were determined by two-way ANOVA, and within one species between treatment—by one-way ANOVA, followed by Holm-Sidak’s post hoc test (α < 0.05) using SigmaPlot (version 3.5, Systat Software Inc., GmbH, Germany). Normality was tested by the Kolmogorov–Smirnov test. If data was not normally distributed, nonparametric tests were applied (e.g., Kruskal–Wallis H test with the Dunn's post hoc test (α < 0.05)).

## Supplementary Information

Supplementary Information.
